# Investigation of the impact of SARS-CoV infection on the immunologic status and lung function after 15 years

**DOI:** 10.1186/s12879-021-06881-3

**Published:** 2021-11-24

**Authors:** Jia Li, Yali Zheng, Lili Zhao, Zhihong Yue, Feng Pan, Yuehong Chen, Bing Yu, Yanwen Chen, Guangyu Zhao, Yusen Zhou, Zhancheng Gao

**Affiliations:** 1 Department of Pulmonary and Critical Care Medicine, Peking University People’s Hospital, Beijing, 100044 China; 2 Department of Clinical Laboratory, Peking University People’s Hospital, Beijing, 100044 China; 3 Department of Radiology, Peking University People’s Hospital, Beijing, 100044 China; 4 Institute of Microbial Epidemiology, Academy of Military Medical Sciences, Beijing, 100039 China

**Keywords:** Severe acute respiratory syndrome (SARS), SARS-CoV specific IgG antibody, Regulatory T cell, HRCT, Pulmonary function test

## Abstract

**Background:**

We investigate the long-term effects of SARS-CoV on patients’ lung and immune systems 15 years post-infection. SARS-CoV-2 pandemic is ongoing however, another genetically related beta-coronavirus SARS-CoV caused an epidemic in 2003–2004.

**Methods:**

We enrolled 58 healthcare workers from Peking University People’s Hospital who were infected with SARS-CoV in 2003. We evaluated lung damage by mMRC score, pulmonary function tests, and chest CT. Immune function was assessed by their serum levels of globin, complete components, and peripheral T cell subsets. ELISA was used to detect SARS-CoV-specific IgG antibodies in sera.

**Results:**

After 15 years of disease onset, 19 (36.5%), 8 (34.6%), and 19 (36.5%) subjects had impaired DL (CO), RV, and FEF_25–75_, respectively. 17 (30.4%) subjects had an mMRC score ≥ 2. Fourteen (25.5%) cases had residual CT abnormalities. T regulatory cells were a bit higher in the SARS survivors. IgG antibodies against SARS S-RBD protein and N protein were detected in 11 (18.97%) and 12 (20.69%) subjects, respectively. Subgroup analysis revealed that small airway dysfunction and CT abnormalities were more common in the severe group than in the non-severe group (57.1% vs 22.6%, 54.5% vs 6.1%, respectively, p < 0.05).

**Conclusions:**

SARS-CoV could cause permanent damage to the lung, which requires early pulmonary rehabilitation. The long-lived immune memory response against coronavirus requires further studies to assess the potential benefit.

*Trial registration* ClinicalTrials.gov, NCT03443102. Registered prospectively on 25 January 2018

## Background

Severe acute respiratory syndrome (SARS) caused by SARS-CoV occurred in China in late 2002 and subsequently spread over the world [1, 2]. Up to September 2003, more than 8000 laboratory-confirmed cases had been documented globally, of which about 30% were severe cases, and 20% were health care workers (HCWs) [3]. At the end of 2019, a new coronavirus (SARS-CoV-2) emerged and caused an outbreak of pneumonia (now called COVID-19) [4]. The novel coronavirus pneumonia has now spread fast all over the world [5] and has infected approximately 11% HCWs [6]. SARS-CoV and SARS-CoV-2 might cause similar pathological changes since they both invade the alveolar epithelial cells through the ACE-2 receptors [7–9]. Both at the SARS-CoV and SARS-CoV-2 in the pulmonary alveolar sac can cause acute respiratory distress syndrome (ARDS) associated with infection of monocytes, macrophages, dendritic cells, and lymphocytes, fluid accumulation in the bronchioles, the release of cytokines, reactive oxygen species, cellular debris, and proteases, further reducing oxygen exchange capacity [10]. In severe COVID-19 infection, the dysregulated immune system responds by secreting cytokines in an uncontrolled manner leading to marked increases in cytokine release, or a “cytokine storm” syndrome [11]. Therefore, we assumed that the long-term effects of SARS-CoV could provide some insight into the impact of COVID-19 on long-term conditions [12]. We investigated the immune system and lung physiology of the patients, 15 years after the SARS-CoV infection Subgroup analyses were conducted to explore the differences between the severe group and the non-severe groups, according to previous severity of illness.

## Methods

### Study design and participants

Fifty-eight HCWs with confirmed SARS-CoV infection in Peking University People’s Hospital in the 2003 pandemic were enrolled in the study. Diagnose criteria were established by the Chinese Centers for Disease Control and Prevention. Fifty-seven of their co-workers, who also had a history of SARS exposure in 2003, were enrolled as controls matched by gender, age, and occupational classifications.

**Fig. 1:**
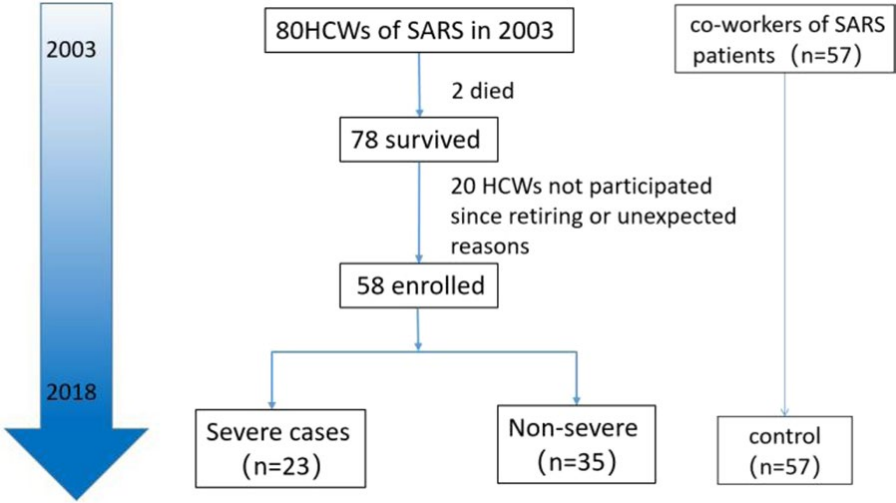
Flow diagram for study participants

The baseline characteristics and medical histories were collected by a standard questionnaire. The evaluation index system in the study includes the general status, the pulmonary status, and the immunity status. The general status was evaluated by regular laboratory tests including complete blood count (CBC) and blood biochemistry examination. Peripheral blood samples were collected from both SARS cases and controls on empty stomach in the early morning. The pulmonary status was assessed by the dyspnea scale (mMRC), the pulmonary function tests (PTFs), and the chest CT scans. The clinical immunologic evaluations of enrolled subjects included quantitation of serum immunoglobulins (IgA, IgG, and IgM) and complement components (C3 and C4), the WBC count, and differential counts of T lymphocyte subsets. Besides, we conducted serologic tests to see whether there are still detectable IgG antibodies against SARS-CoV after 15 years. The flowchart is shown in Fig. 1. Specific steps for the process are as follows.

### SARS-CoV IgG antibody test

Enzyme-linked immunosorbent assay (ELISA) was performed to detect the IgG antibodies against SARS-CoV in serum. For IgG detection, ELISA plates were coated with purified recombinant SARS protein antigens (S-RBD protein or N protein). Serum samples (diluted 1:160) and negative and positive controls were added to the wells of the coated plates in a total volume of 100 μl, plates were then incubated at 37 °C for 30 min. After five wash steps with washing buffer, 100 μl of diluted HRP-conjugated anti-human IgG antibodies was added to the wells, and samples were incubated at 37 °C for 30 min. After five wash steps with washing buffer, 50 μl of TMB substrate solution and 50 μl of the corresponding buffer were added, and samples were incubated at 37 °C for 10 min. The reaction was terminated by adding 50 μl of 2 M sulfuric acid, and A450 was measured.

Sixteen serum samples from a pneumonia cohort (NCT 03093220) were used as controls to rule out the nonspecific cross-immunoreactivity. Absorbance at 450 nm was measured by ELISA Microplate Reader. The cut-off OD value was calculated as 1.5 times the normal threshold of the controls.

### Lung imaging and interpretation

A total of 55 patients underwent lung high-resolution CT (HRCT) examination in March 2018. HRCT was performed by the GE 256-row Revolution scanner with inspiratory and expiratory phases, respectively. The radiological images were reviewed independently by two radiologists and final interpretation was established by consensus, including the following HRCT abnormalities: ground-glass opacity, consolidation, fibrous strip shadow, emphysema, pulmonary bullae, bronchiectasis, and pleural thickening.

### Pulmonary function tests (PFTs)

Dyspnea in daily living was evaluated by the mMRC scale. Pulmonary function tests (PFTs) included spirometry, plethysmography, diffusing capacity of carbon monoxide were conducted. The main observation indicators included total lung capacity (TLC), residual volume (RV), forced vital capacity (FVC), forced expiratory volume in one second (FEV_1_), one-second rate (FEV_1_/FVC), maximum mid-expiratory flow rate 25–75 (FEF_25–75_), and carbon monoxide diffusion amount [DL (CO)] measured by a single breath test. Values below 80% prediction were regarded as being impaired. RV over 120% predicted and FEF_25–75_ below 65% predicted as being damaged.

### Statistical analysis

All the analyses were performed with the SPSS software, version 22.0, (IBM Corp., Armonk, NY, USA) unless otherwise indicated. Continuous variables were expressed as medians (interquartile ranges) or mean ± SD and compared by Student’s t-test or Mann–Whitney U tests for normal or non-normal distribution, respectively. Categorical variables were expressed as numbers (proportion). p-values < 0.05 were considered significant.

### Ethical approval

The study was approved by the ethics committee of Beijing University People’s Hospital (PHB0102018-01) and was registered on the Clinical trials website (https://clinicaltrials.gov/) (NCT03443102). The written informed consent was obtained from all participants.

## Results

### Demographic and clinical features

As shown in Table 1, among the 58 HCWs enrolled in the study, ten were male (17.2%), and the median age was 46 years (IQR 40.0–50.5) up to 2018. The majority of them were nurses who worked in the Emergency Department during the 2003 pandemic. Thirty-five patients were categorized as non-severe pneumonia in 2003, and 23 cases as severe pneumonia. Up to March 2018, most of them (47/58, 81.0%) were still full-time employed. Sixteen of them had hypertension, five had diabetes, and one had Sjogren’s syndrome. Nearly half of the subjects complained of fatigue (27/58, 46.55%). Thirty-nine (39/56, 69.6%) SARS survivors had an mMRC score of 0–1 and 17 subjects (17/56, 30.4%) had 2–3 (Table 2).

**Table 1:** Demographic data of SARS survivors and controls

	The severe group (N = 23)	The non-severe group (N = 35)	The control group (N = 57)	F/T value	P-value
Age of onset					
< 30 years	6 (26.1%)	18 (51.4%)	28 (49.1%)	4.294	0.117
≥ 30 years	17 (73.9%)	17 (48.6%)	29 (50.9%)		
Gender					
Female	19 (82.6%)	29 (82.9%)	46 (80.7%)	0.082	0.960
Male	4 (17.4%)	6 (17.1%)	11 (19.3%)		
Occupations					
Doctor	3 (13.0%)	4 (11.4%)	16 (28.1%)	9.079	0.169
Nurse	15 (65.2%)	25 (71.4%)	32 (56.1%)		
Technician	3 (13.0%)	6 (17.1%)	8 (14.0%)		
Others	2 (8.7%)	0	1 (1.8%)		
Departments					
Emergency room	11 (47.8%)	12 (39.3%)	26 (45.6%)	4.871	0.771
Cardiology	3 (13.0%)	3 (8.6%)	3 (5.3%)		
Pulmonology	2 (8.7%)	2 (5.7%)	7 (12.3%)		
Surgery	2 (8.7%)	3 (8.6%)	3 (5.3%)		
Others	5 (21.7%)	15 (42.9%)	18 (31.6%)

**Table 2:** The lung status of SARS survivors assessed by mMRC score, pulmonary function tests, and chest CT

	The severe group	The non-severe group	F/T	P
mMRC score^a^	N = 22	N = 34		
0	5 (22.7%)	11 (32.4%)	4.785	0.310
1	11 (50.0%)	12 (35.3%)		
2	3 (13.6%)	10 (29.4%)		
3	3 (13.6%)	1 (2.9%)		
Pulmonary function parameters^b^	N = 21	N = 31		
FVC (L)	3.10 ± 0.59	3.52 ± 0.6	− 2.517	0.015
FVC (%)	101.41 ± 14.52	104.69 ± 13.30	0.835	0.408
FVC% < 80%	0	0	–	–
FEV1(L)	2.48 ± 0.514	2.90 ± 0.453	3.313	0.003
FEV1 (%)	95.21 ± 12.46	101.61 ± 12.66	1.604	0.108
FEV1% < 80%	4 (19.0%)	0	6.397	0.011
FEV1/FVC (%)	79.23 ± 4.35	82.64 ± 4.80	2.611	0.012
FEV1/FVC < 70%	1 (4.8%)	0	1.505	0.220
DLCO (mmol/min/KPa)	6.86 ± 1.31	7.18 ± 1.43	0.807	0.424
DLCO (%)	84.01 ± 11.06	82.18 ± 12.42	− 0.563	0.576
DLCO% < 80%	6 (28.6%)	13 (41.9%)	0.964	0.326
TLC (L)	5.05 ± 0.87	5.39 ± 0.71	1.599	0.116
TLC (%)	100.32 ± 10.83	103.93 ± 9.37	1.277	0.207
TLC% < 80%	0	0	–	–
RV/TLC (%)	38.69 ± 5.86	37.23 ± 5.77	− 0.889	0.378
RV (%)	107.73 ± 13.66	111.70 ± 17.22	0.884	0.381
RV% > 120%	6 (28.6%)	12 (38.7%)	0.569	0.451
FEF25–75 (%)	65.95 ± 19.73	82.69 ± 20.36	2.946	0.005
FEF25–75% < 65%	12 (57.1%)	7 (22.6%)	6.449	0.011
CT abnormalities^c^	N = 22	N = 33		
GGO	6 (27.3%)	2 (6.0%)	16.360	< 0.001
Interstitial fibrosis	7 (31.8%)	0		
Others	5 (22.7%)	1 (3.0%)		

a, b, c, The number of SARS survivors undertaking different tests

### Immunological features 15 years after recovering from the SARS-CoV pneumonia

The results of CBC and biochemical tests were within the normal range, as well as serum immunoglobulin tests. There was no statistically significant difference among the severe group, the non-severe group, and the control group (p > 0.05). Complement 4 (C_4_) were a bit higher in the non-severe group, with values of 0.22 ± 0.06, 0.27 ± 0.18 and 0.22 ± 0.06 g/L, respectively, F = 3.547, p = 0.032. (Table 3).

**Table 3:** Laboratory evaluation of immune status of SARS survivors

	The severe group (N = 23)	The non-severe group (N = 35)	The control group (N = 57)	F/T value	P-value
IgA (G/L)	2.20 ± 0.97	2.41 ± 0.97	2.21 ± 0.73	0.555	0.576
IgG (G/L)	11.84 ± 1.97	12.80 ± 2.32	12.94 ± 2.18	2.985	0.055
IgM (G/L)	0.98 ± 0.40	1.03 ± 0.38	1.09 ± 0.45	0.742	0.478
C3 (G/L)	0.88 ± 0.13	0.98 ± 0.18	0.94 ± 0.18	2.928	0.058
C4 (G/L)	0.22 ± 0.06	0.27 ± 0.18	0.22 ± 0.06	3.547	0.032
CD4 + (%)	50.69 ± 13.21	51.82 ± 10.25	51.20 ± 10.10	0.071	0.931
CD8 + (%)	40.82 ± 10.97	39.71 ± 10.61	38.48 ± 9.75	0.569	0.568
CD4 + CD25 + Foxp3 + (%)	3.84 ± 1.43	3.89 ± 1.12	3.34 ± 0.97	2.862	0.061

*IgA* immunoglobulin A, normal value 0.82–4.53 g/L, *IgG* immunoglobulin G, normal value 7.2–16.8 g/L, *IgM* immunoglobulin M, normal value 0.46–3.04 g/L, *C3* complement3, normal value 0.79–1.52 d/L, *C4* complement4, normal value 0.16–0.38

**Fig. 2:**
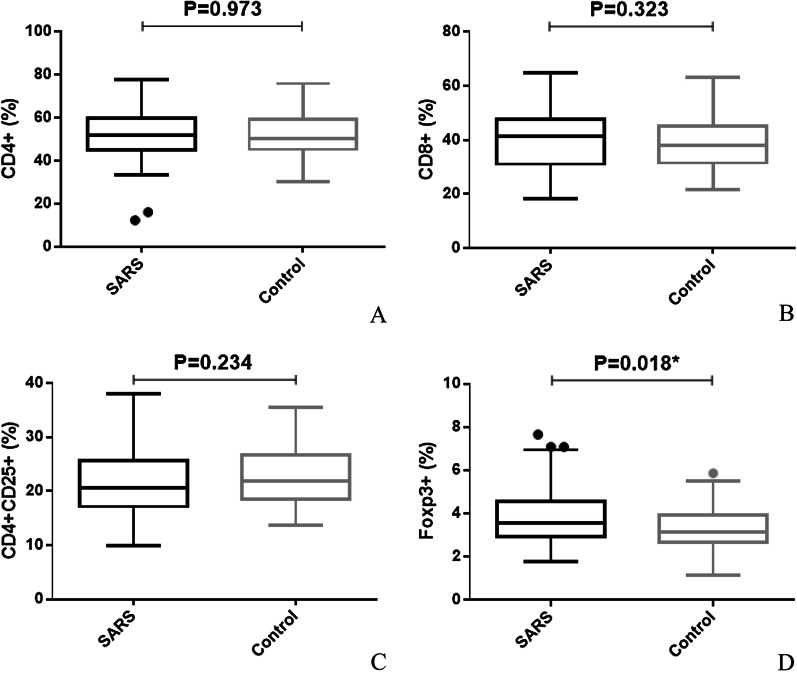
Peripheral T cell subsets between SARS survivors and healthy controls. The proportion of CD4 + (**A**), CD8 + (**B**), CD4 + CD25 + (**C**) and CD4 + CD25 + Foxp3 + regulatory T cells (**D**) between SARS survivors and healthy controls

T cell subsets were all within normal limits. The percentage of peripheral CD_4_^+^ CD_25_^+^ Foxp_3_^+^ regulatory T cells was slightly higher in the SARS survivors, as compared to the controls (3.85 ± 1.31% vs 3.34 ± 0.97%, respectively, T = 2.420, p = 0.018) (Fig. 2).

**Fig. 3:**
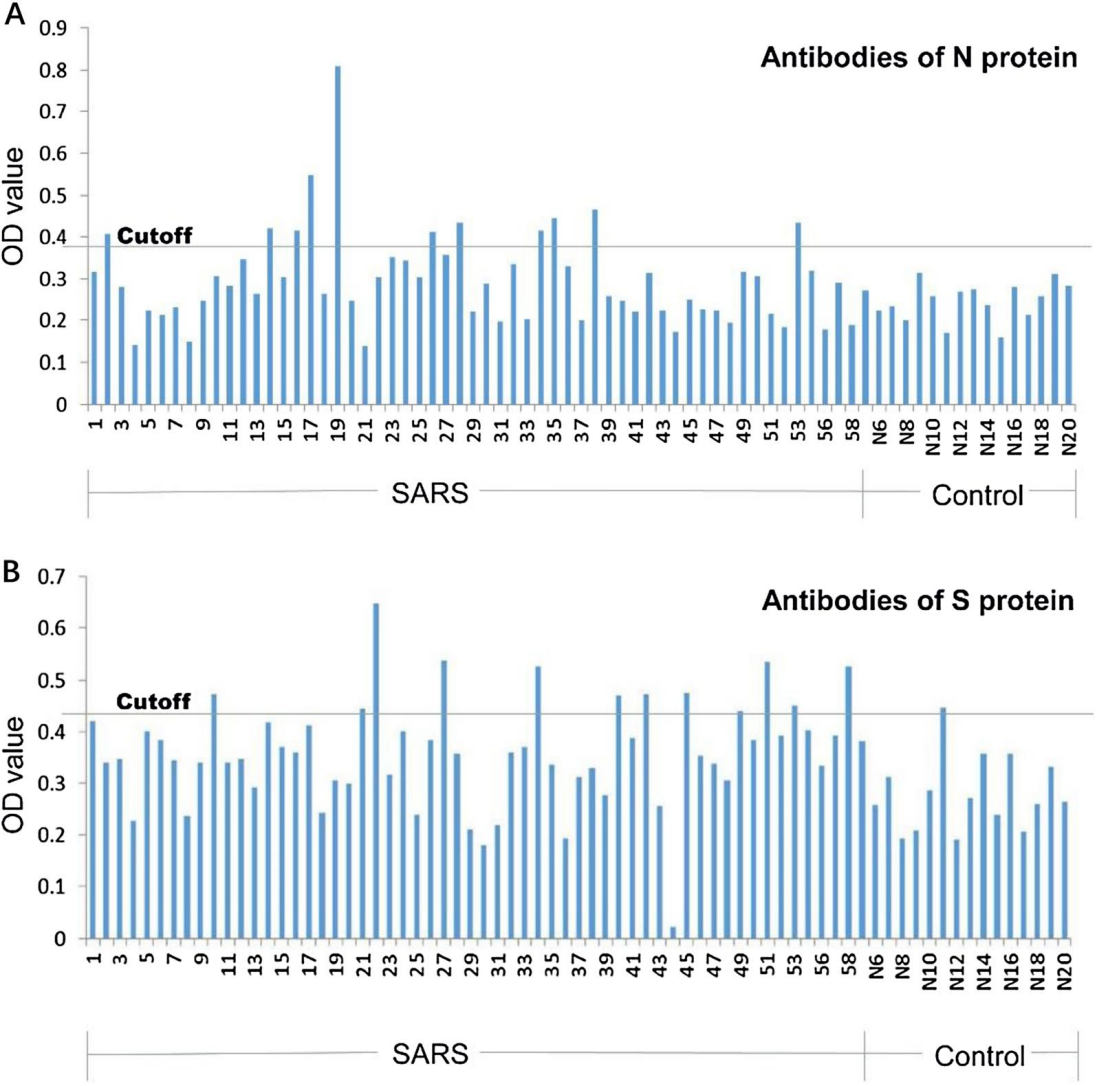
SARS-CoV specific IgG antibodies in serum after 15 years of infection. ELISA results of Specific IgG antibody against SARS N protein (**A**) and against S-RBD protein (**B**). *S-RBD* receptor binding domain of spike protein, *N protein* nuclear protein

The serum antibody test results of SARS survivors showed that SARS-CoV N protein IgG antibody and S-RBD protein IgG antibody were positive in 11 (18.97%) and 12 (20.69%), respectively (Fig. 3).

### Lung imaging results

**Fig. 4:**
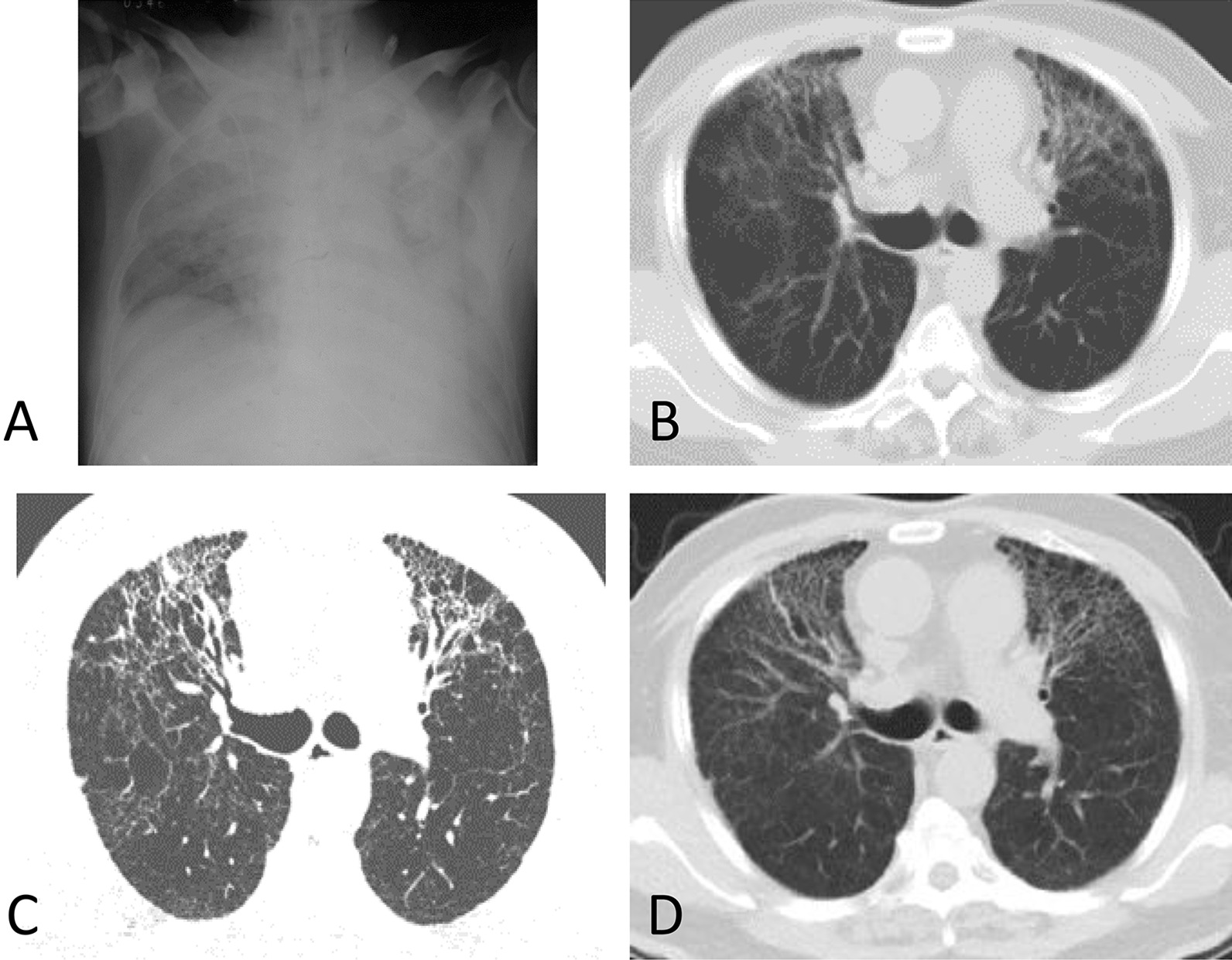
Serial CT images of a male SARS survivor in 15 years. **A** Showed diffused consolidation in bilateral lungs on June 14, 2003. **B**–**D** Showed a stable reticular interstitial fibrosis with tractive bronchiectasis in the serial follow-up, after 1 year (**B**, November 2004), 3 years (**C**, August 2006) and 15 years (**D**, March 2018) of SARS pneumonia

CT abnormalities including ground-glass opacities, interstitial fibrosis, emphysema, bullae, and pleural thickening were observed in 14 SARS survivors, mostly (12/14, 85.6%) in the severe group. The most common abnormal lung radiologic findings were ground-glass opacities, detected in 8 (57.1%) cases. Seven cases presented with interstitial fibrosis. Less common changes included emphysema, bullae, and pleural thickening, and none of the survivors showed pleural effusion. We compared longitudinal series of chest CT in some cases of the severe group, and the abnormalities remained relatively stable 1 year after the disease onset. Dynamic CT changes of a patient in the severe group were shown in Fig. 4.

### Pulmonary function tests

Pulmonary function tests including spirometry, lung volume and diffusion capacities proceeded in 52 subjects. One severe case had moderate impairment of FVC (1/52, 1.92%), and no one showed restrictive ventilatory dysfunction. The FEF_25–75%_ decreased in 19 patients (36.54%) with the mean (SD) 75.93 ± 21.57%. Diffusion capacity was impaired in 19 patients (36.54%), and the lowest was 62.2% of predicted values.

FEV_1_, FEV_1_/FVC and FEF_25–75%_ were lower in the severe group than that in the non-severe group (FEV_1_2.48 ± 0.514% vs 2.90 ± 0.453L, FEV_1_/FVC 79.23 ± 4.35% vs 82.64 ± 4.80%, FEF_25–75%_ 65.95 ± 19.73% vs 82.69 ± 20.36%, p = 0.003, 0.012, and 0.005, respectively). There was no statistically significant difference in lung diffusing capacity of carbon monoxide between the two groups (84.01 ± 11.06% vs 82.18 ± 12.42%, p = 0.576). All the results of PFTs are provided in Table 2.

## Discussion

We investigated the current status of convalescent SARS patients to find out whether there is any long-term effect of SARS-CoV infection. In our study, most cases have continued to normal operation as expected. The majority of PFT values, cellular and humoral immune parameters are within the normal range. However, cases in the severe group revealed poor lung function than cases in the non-severe group, including small airway function (FEF_25–75_%) and diffuse capacity [DL (CO)]. Plus the persistent CT abnormalities, our study indicated that SARS-CoV can cause permanent damage to the lung, especially in critically ill patients. Besides, the high percent of Treg cells in peripheral blood and the durability of serum antibodies indicate a persistent immune memory to SARS-CoV in both T- and B- cells. However, whether it is a virus-specific protective immune response requires further exploration.

Our study found that the pulmonary functions of SARS survivors largely returned to normal after 15 years of convalescence, most of them (81.03%) were still full-time employed, and 69.6% had an mMRC scale score of 0–1. The lung function damage was characterized by small airway abnormalities (36.5%) and reduced diffusion capacity (36.5%). Previous studies evaluating the lung function status [13, 14] in SARS survivors observed consistent results with our study. The impairment of DL (CO) was significant in all studies, which suggests impairment in the intra-alveolar diffusion pathway. Airway obstruction and small airway dysfunction were less common in the studies. In our study, small airway dysfunction was as common as damaged diffusion capacity. Survivors from severe COVID-19 also revealed small airway dysfunction [15, 16]. As previously reported, the predominant pathological finding in SARS was diffuse alveolar damage in the early phase of the disease [17] but in the later course of the disease, dense septal and alveolar fibrosis were seen [18]. Recently report demonstrated that the COVID-19 virus particles could be observed in distal airway mucosal epithelial through electron microscopy [19]. Therefore, bronchiolitis may exist and result in dysfunction of the small airway.

No statistical differences in the pulmonary function measurements were observed in the longitudinal studies [13, 14], as compared with those measured either immediately after recovery or after 24 months post-illness. These results are in accord with our study, which showed persistent CT abnormalities in SARS convalescent patients. Besides, we previously reported that the CT abnormalities remained stable after 24 months post-infection [20]. These data suggested permanent lung damage due to coronavirus and indicated the necessity of early respiratory rehabilitation training.

We also found that approximately 20% of SARS convalescent patients still have a detectable level of serum IgG specific to SARS-CoV, with 20.69% positive to S-RBD protein and 18.97% positive to N protein, which was not related to the disease severity. These findings are somewhat surprising given the fact that other researches have shown that SARS-CoV-specific antibodies waned over time [21] and SARS-CoV-specific IgG antibodies could not be detected in more than 90% of patients six years after infection [22]. The researchers have reported long-lived SARS-CoV-specific memory T cells but not B cells in SARS survivors. SARS-CoV reactive CD4 + T cells could be detected even 17 years after infection [23]. Our data indicated the existence of virus-specific memory B cells in the peripheral blood, which could still be activated to secret IgG up to 15 years post-infection. An improved detection threshold might explain the different results. Further evidence is required to validate whether the antibodies can confer protection against SARS-CoV, or even better, cross-protection against COVID-19.

Besides, we observed a slight elevation of Treg cells in peripheral blood in SARS survivors after 15 years, as compared with healthy controls (3.85 ± 1.31% vs 3.34 ± 0.97%). The values were still within the normal range and no clinical features were observed to be related to the higher proportion of Treg cells, including the disease severity. However, the autoimmune characteristics have been observed in critical COVID-19 cases [24], and autoimmune and inflammatory diseases were observed following COVID-19 as well [25]. It is well known that Treg-mediated active maintenance of self-tolerance is the pathogenetic mechanism of autoimmune diseases [26]. Although the connections between SARS and autoimmune or inflammatory diseases were not reported, the cross-reaction of SARS-CoV antigen with autoantibodies had already been observed as early as 2004 [27]. In addition to this, Treg cells can also limit pulmonary immunopathology in respiratory virus infections [28]. And in West Nile infection, Tregs can protect from central nervous system immunopathology by controlling CD8 T-cell responses [29]. Brandon Malone etc. had recently revealed the diversity of the immunogenic landscape of SARS-CoV-2 among the different HLA groups [30]. The exact role of Treg cell reaction to coronavirus infection might need to be further explored.

There are several limitations to this study. First, although this study had the largest sample size for an evaluation of the long-term effects of SARS, we failed to make serial assessments over 15 years. This limitation means that study findings need to be interpreted cautiously. Further comparisons over time would be required to verify the observations. Second, although full lung function tests were conducted in our study, we did not perform 6MWT or cardiopulmonary exercise testing to verify their exercise capacities, as a great part of our cases were complaining of fatigue when questioned. The results might therefore not be representative of the entire cohort, especially those with normal DL (CO). Additional factors such as muscle deconditioning and steroid myopathy should be considered in further studies.

## Conclusions

In summary, more than 30% of SARS survivors had impaired DL (CO) and over 30% of them had small airway dysfunction 15 years after SARS onset. CT abnormalities remained in over 20% of SARS subjects. SARS can lead to persistent lung abnormalities in survivors. Health authorities should provide more support for early pulmonary rehabilitation. The long-lived immune memory response requires further research to assess the potential beneficial immunity against coronavirus.

## Data Availability

The datasets used and/or analyzed during the current study are available from the corresponding author on reasonable request.

## Abbreviations

ACE2: Angiotensin-converting enzyme 2
CoV: Coronavirus
DIC: Disseminated intravascular coagulation
DLCO: Diffusion capacity for carbon monoxide of the lung
DLCO/VA: Diffusion capacity for carbon monoxide of the lung/Alveolar volume
ELISA: Enzyme-linked immunosorbent assay
FEF_25–75%_: Forced expiratory flow between 25 and 75% of forced vital capacity
FEV_1_: Forced expiratory volume in one second
FEV_1_/FVC%: Forced expiratory volume in one second/Forced vital capacity
FVC: Forced vital capacity
GGO: Ground-Glass opacity
HCWs: Health care workers
HRCT: High-resolution computerized tomography
HRQoL: Health-related quality of life
RT-PCR: Reverse transcription polymerase chain reaction
RT-PCR: Real-time PCR
RV: Residual volume
SARS: Severe acute respiratory syndrome
TLC: Total lung capacity
WHO: World Health Organization
